# Single Nucleotide Polymorphism *rs17173608* in the Chemerin Encoding Gene: Is It a Predictor of Insulin Resistance and Severity of Coronary Artery Disease in Non-Obese Type 2 Diabetes?

**DOI:** 10.3390/healthcare9060623

**Published:** 2021-05-23

**Authors:** Sangeetha Perumalsamy, Wan Azman Wan Ahmad, Hasniza Zaman Huri

**Affiliations:** 1 Department of Clinical Pharmacy & Pharmacy Practice, Faculty of Pharmacy, University of Malaya, Kuala Lumpur 50603, Malaysia; geetha2_20@yahoo.com; 2 Cardiology Unit, Department of Medicine, Faculty of Medicine, University of Malaya, Kuala Lumpur 50603, Malaysia; wanazman@ummc.edu.my; 3 Clinical Investigation Centre (CIC), University Malaya Medical Centre, Petaling Jaya 59100, Malaysia

**Keywords:** chemerin, *RARRES2*, *rs17173608*, insulin resistance, T2D, severity of CAD

## Abstract

(1) Background: Chemerin, or the *RARRES2* (*Retinoic Acid Receptor Responder 2*) gene, is found to be associated with an increased incidence of insulin resistance, endothelial dysfunction, type 2 diabetes (T2D), and coronary artery disease (CAD). This study investigates associations of *RARRES2* *rs17173608* with insulin resistance and the severity of CAD in non-obese T2D patients in relation to the clinical and genetic factors. (2) Methods: A total of 300 patients with T2D and CAD were recruited in this study. The associations of insulin resistance and the severity of CAD with *RARRES2* *rs17173608* and clinical factors were assessed. The genotyping procedures were performed using the TaqMan method. The significant associations (*p* ≤ 0.05) from preliminary tests were employed to carry out the secondary analysis. (3) Results: *RARRES2* *rs17173608* (TT, TG, and GG polymorphisms in the preliminary analysis; TG and GG polymorphisms in a secondary analysis) was associated with insulin resistance and the severity of CAD in both the preliminary and secondary analysis (all *p*-values were < 0.05). Additionally, in the secondary analysis, FPG and ACEI were also associated with insulin resistance and the severity of CAD (all *p*-values were < 0.05). (4) Conclusion: From the preliminary findings, *rs17173608* is a significant predictor of insulin resistance and the severity of CAD.

## 1. Introduction

Chemerin, also known as *retinoic acid receptor responder protein 2 (RARRES2)* and *tazarotene50 induced gene 2* [1], is a chemoattractant protein involved in the pathogenesis of metabolic syndrome [2]. It is secreted in the liver, acts as a chemotactic agent, and is highly stimulated by elements of the innate immune system, such as plasmacytoid dendritic cells and macrophages [2]. Chemerin induces angiogenesis of endothelial cells and results in endothelial dysfunction [3]. Besides which, it can activate inflammatory response and oxidative stress in adipose tissue, results in insulin resistance and further enhance endothelial dysfunction [4]. Endothelial dysfunction and insulin resistance cause the progression of type 2 diabetes (T2D) and atherosclerotic coronary artery disease (CAD).

The CAD is classified as severe when the atherosclerotic plaques narrow down the vessels with stenosis by more than 50% [5]. In a study by Dahpy et al., serum chemerin levels were higher in patients with T2D and metabolic syndromes than nondiabetics and patients without metabolic syndromes [4]. There is growing evidence showing a relationship between coronary atherosclerosis and chemerin. Several cross-sectional studies showed an independent correlation between chemerin concentrations and CAD. Chemerin is moderately heritable, with 16–25% of variations ascribed to genetic factors [5].

The encoding gene of chemerin, *RARRES2*, is also associated with an increased incidence of metabolic syndrome and inflammatory diseases [6]. Metabolic syndrome independently predicts the development of T2D and CAD [7]. T2D patients have a higher cardiovascular morbidity and mortality rate. The diabetic vascular disease causes a two–fourfold increase in CAD occurrence [8]. In Malaysia, 3.6 million people have diabetes, and most of them have T2D [9]. Meanwhile, CAD is the leading cause of death in T2D patients in Malaysia [10]. However, the relationships between *RARRES2*, inflammation, metabolic syndromes, insulin resistance, and CAD have not been completely elucidated.

Genetic polymorphisms can play an essential role in phenotypic variation disease susceptibility [11]. Studies show that the single nucleotide polymorphisms (SNPs) of the *RARRES2* gene is associated with an increased incidence of T2D and CAD [12,13]. The *RARRES2 rs17173608* SNP had been associated with metabolic syndromes and obesity. It is located in the potential site of mutation, intron 2 of *RARRES2* [14,15]. However, the literature on the association of *rs17173608* with insulin resistance and the severity of CAD in T2D patients is lacking. In a previous study conducted by Mehanna et al., the minor G allele of *rs17173608* SNP showed its significant association with metabolic syndromes in Egyptian women [16]. In another study, the TT genotype of *rs17173608* was more widely distributed in T2D patients than non-diabetics [4]. In this study, we evaluated the association *rs17173608* with insulin resistance and the severity of CAD in Malaysian T2D patients for the first time.

## 2. Methods and Materials

### 2.1. Participants

We conducted a cross-sectional study with 300 patients from the diabetic clinic and cardiology clinics of University Malaya Medical Centre (UMMC) after the approval of the UMMC Medical Ethics Committee (ref number: 20158-1552). Written, informed consent was obtained from the participants before the blood samples were taken. For analysis, the patients were divided into three groups; T2D patients with CAD (*n* = 150), patients with T2D only (*n* = 90), and patients with CAD only (*n* = 60). The T2D patients with CAD group were the case group of this study, whilst the other two were the control groups. We excluded patients with type 1 diabetes, critically ill patients, asthma patients, and obese patients (more than 30.0 kg/m^2^) [15].

### 2.2. Sample Size Calculation

Quanto software version 1.2.4 was used to calculate the sample size (USC, Los Angeles, CA, USA). The sample size for detecting the association between disease and SNP marker was positively affected by disease prevalence, disease allele frequency, and inheritance model. According to the National Cardiovascular Disease Database, only about 20% of CAD patients have T2D [17]. At the same time, we had very stringent inclusion and exclusion criteria. We only included patients that had stable CAD with more than 50% stenosis. We excluded obese patients as obesity may influence the outcome of the study, as *rs17173608* had been associated with obesity in previous studies [4,15]. Thus, the sample size is smaller. From the calculation, the sample size was 300 (case (T2D patients with CAD patients) = 150 + controls (T2D only patients + CAD only patients) = 150, Total = 300) (Table 1).

### 2.3. Demographic and Clinical Information

The demographic factors, such as gender, race, and family history of T2D and CAD, were obtained from the electronic medical record system (EMR) of University Malaya Medical Centre (UMMC) and the National Cardiovascular Disease Database (NCVD). Age was calculated based on the patients’ birth years. The EMR also provided information on the types and number of comorbidities, pharmacological treatments and concomitant drugs, and the duration of T2D and CAD.

### 2.4. Anthropometric Measurement

The anthropometric parameters of height and weight were obtained for each participant. BMI (kg/m^2^) was calculated by dividing weight by the square of the height [18].

### 2.5. Biochemical Parameters

After at least eight hours of fasting, blood samples were collected. A YSI 7100 multiparameter bioanalytical system [19] was used to analyse the plasma glucose concentrations. In the meantime, plasma insulin concentrations were determined using the Santo Advia Centaur XP immunoassay system [20]. The Beckman Coulter Immage 800 immunochemistry system was used to measure high-sensitivity C-reactive protein (hs–CRP) levels, and the G8 HPLC analyzer was used to measure A1C concentrations [21]. Triglyceride (TG), total cholesterol (TC), and high-density lipoprotein cholesterol (HDL-c) levels were measured using an automated analyser, the Dimension^®^ RxL Max^®^ integrated chemistry system [22]. The homeostasis model assessment—insulin resistance (HOMA-IR) was calculated by multiplying fasting plasma insulin (FPI) and fasting plasma glucose (FPG) and dividing by 22.5 [23]. Serum chemerin concentrations were measured using an ELISA kit and read at 450 nm using a microplate reader [24].

### 2.6. Selection of SNP

The candidate gene approach was used to discover the SNP. Initially, risk variants and candidate SNPs associated with T2D and CAD were identified. The putative SNPs were then identified by investigating their relevance to the disease mechanism. The selected SNPs were then evaluated by identifying existing gene variants and determining which variants result in proteins with altered functions that may influence the trait of interest [25], with *rs17173608* being chosen because it had an intronic functional consequence on gene regulation. The *rs17173608* variant is located on the second intron of the chemerin gene and has previously been linked to T2D and CAD [26].

### 2.7. Genetic Analysis

The Qiagen DNA blood mini kits (QIAGEN N.V., Venlo, The Netherlands) were used for DNA extraction. The DNA was quantified using a NanoDrop ND-1000 spectrophotometer (Thermo Fisher Scientific, Waltham, MA, USA). Polymorphism *rs17173608* was analysed using real-time polymerase chain reaction using the TaqMan fluorogenic 5′ nuclease assay. The StepOnePlusTM real-time PCR system assay (Applied Biosystems, Foster City, CA, USA) was used to amplify the polymorph. Applied Biosystems StepOnePlusTM real-time software was used to compute the results (Thermo Fisher Scientific, Waltham, MA, USA).

### 2.8. Statistical Analysis

The IBM Statistical Package for the Social Sciences (SPSS) (Armok, NY, USA) software version 26.0 was used to analyse the data. Descriptive statistics, expressed as frequency, were used to summarise patients’ demographic and clinical characteristics (*N*, %). Continuous variables were summarised as mean ± SD (normally distributed) or median ± IQR (not normally distributed). Chi-square and Fisher exact test were used to study the differences in categorical variables. The significance of all continuous demographics and biochemical parameters among the groups was assessed using one-way ANOVA and the Kruskal–Wallis test. HWE analyses were applied to compare the observed and expected genotype frequencies of subjects by a goodness-of-fit chi-square using the genepop software (http://genepop.curtin.edu.au) (accessed on 5 January 2021) [27]. For the HWE test, P-values greater than 0.05 were deemed significant. Binary and multinomial logistic regression tests (univariate and multivariate) were employed in the preliminary and secondary analysis, after which the age, race, BMI (Body Mass Index), and gender were adjusted as covariates.

Binary logistic regression was used to analyse the association model of insulin resistance with *rs17173608* and clinical factors. Meanwhile, multinomial logistic regression analysis was used to assess the association model of the severity of CAD with *rs17173608* and clinical factors. The preliminary analysis’s significant associations (*p* ≤ 0.05) were used to carry out the secondary tests. The cut-off point of insulin resistance was determined using a plotted receiver-operating characteristic (ROC) curve. The point on the ROC curve with maximum Youden index (sensitivity-(1-specificity)) and the point with the shortest distance from the point (0, 1) ((1-sensitivity)2 + (1-specificity)2) were calculated to find the optimal threshold of HOMA-IR and a cut-off point of 7.17 obtained [28].

### 2.9. Operational Definitions

- Insulin resistance: HOMA-IR value of more than 7.17 from the ROC curve is categorised as insulin resistance (IR) whilst less than 7.17 is categorised as insulin-sensitive (IS).
- The severity of CAD: one, two, or three arteries narrowed by 50% of stenosis. One vessel = single-vessel disease (SVD), two vessels = double-vessel disease (DVD), three vessels = triple-vessel disease (TVD). TVD is the most severe type of vessel disease (3 major vessels involved) [5].
- Obesity: BMI more than or equal to 30 kg/m^2^ [15].
- Preliminary analysis: one-way ANOVA, Chi-square, Fisher exact test, Kruskal–Wallis test, one-sample T-test, binary logistic regression (univariate tests), multinomial logistic regression (univariate tests).
- Secondary analysis: binary logistic regression (multivariate tests), multinomial logistic regression (multivariate tests).
- Univariate tests: *p* values were obtained by running binary/multinomial analysis for each variable separately.
- Multivariate tests: involved a set of multiple variables that were significant from the univariate tests. *p* values were obtained for each of the variables involved by running the binary/multinomial analysis as a group.

## 3. Results

### 3.1. Demographic and Clinical Data

Table 2 shows the demographic and clinical data of the study population. The number of males was higher in T2D patients with CAD, and CAD-only patients. The average age of patients was higher in the CAD-only group compared to the other two groups. Indian patients were more prone to T2D with or without CAD, in comparison with Malay and Chinese patients. Patients with only CAD had a lower BMI than T2D patients with CAD and T2D-only patients. Most of the patients had a family history of T2D and CAD.

### 3.2. Hardy–Weinberg Equilibrium Test for SNP rs17173608

The SNP *rs17173608* did not deviate from the Hardy–Weinberg Equilibrium (*p* > 0.05) in T2D patients with CAD (*p* = 0.98), T2D-only (*p* = 0.92), and CAD-only (*p* = 0.22) groups. Thus, the SNP was included in the subsequent analyses.

### 3.3. Associations of Clinical Factors with rs17173608

From the preliminary statistical analysis, among the OHA- and insulin- combination treatments, biguanides and insulin were associated with *rs17173608*. Among the mono-therapy treatments, biguanides were associated with *rs17173608* in T2D patients with CAD. Sulphonylureas and CCBs were found to be associated with *rs17173608* in the T2D-only group. Meanwhile, FPG, FPI, and A1C levels were significantly associated with *rs17173608* in CAD-only groups. Among all these significant associations, the association of FPG with *rs17173608* was the strongest, with an OR value of 6.053. Table 3 demonstrates the associations of clinical factors with *rs17173608* in the study population.

### 3.4. Associations of rs17173608 with Insulin Resistance and Severity of CAD

**Figure 1:**
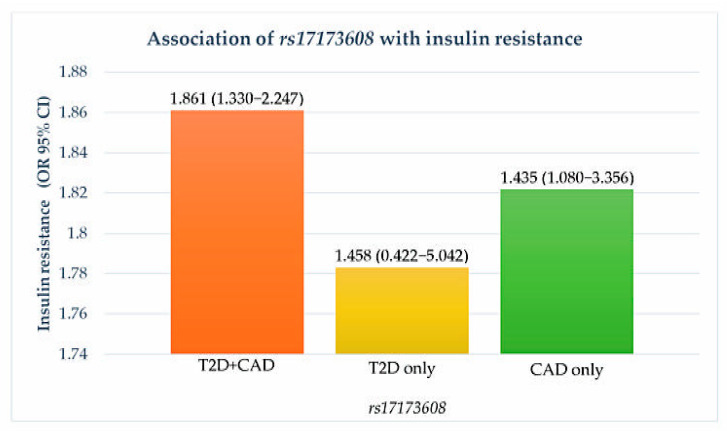
Association of *rs17173608* with insulin resistance. OR: odds ratio; CI: confidence interval.

**Figure 2:**
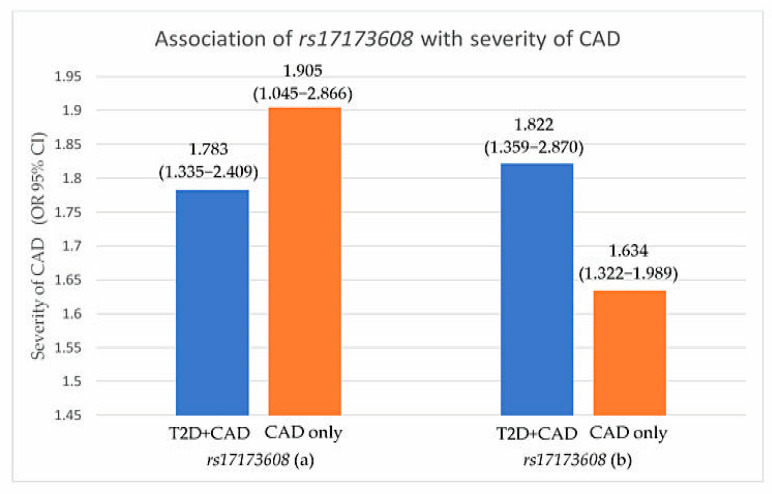
Association of *rs17173608* with severity of CAD. (**a**) SVD vs. DVD, (**b**) SVD vs. TVD. OR: odds ratio; CI: confidence interval.

Preliminary associations of *rs17173608* with insulin resistance and the severity of CAD are presented in Table 4, Figure 1 and Figure 2. The preliminary results showed significant correlations between *rs17173608* and insulin resistance and the severity of CAD in T2D patients with CAD and CAD-only groups. Secondary analysis using binary and multinomial logistic regression was carried out to find out the associations.

### 3.5. Association Models of Insulin Resistance and Severity of CAD in Correlation with rs17173608 and Clinical Factors (Secondary Analysis)

**Figure 3:**
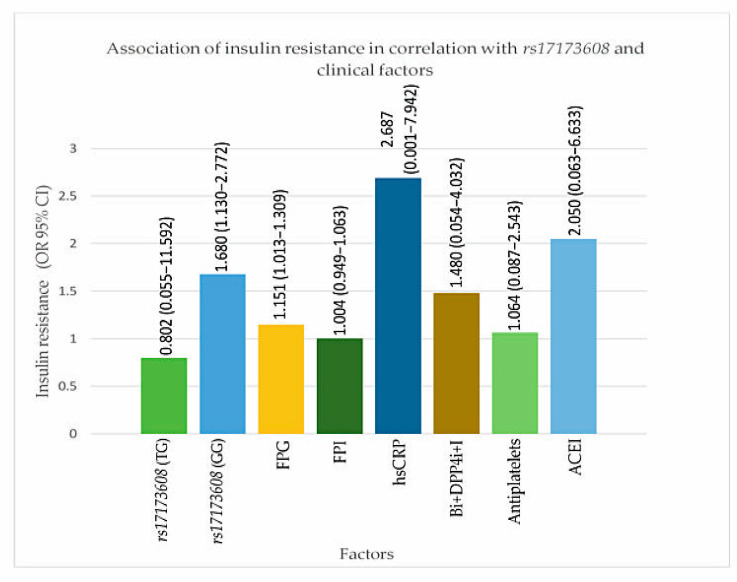
Association of insulin resistance in correlation with *rs17173608* and clinical factors. FPG: Figure 4. i: dipeptidyl peptidase-4 inhibitor; I: insulin. OR: odds ratio; CI: confidence interval.

**Figure 4:**
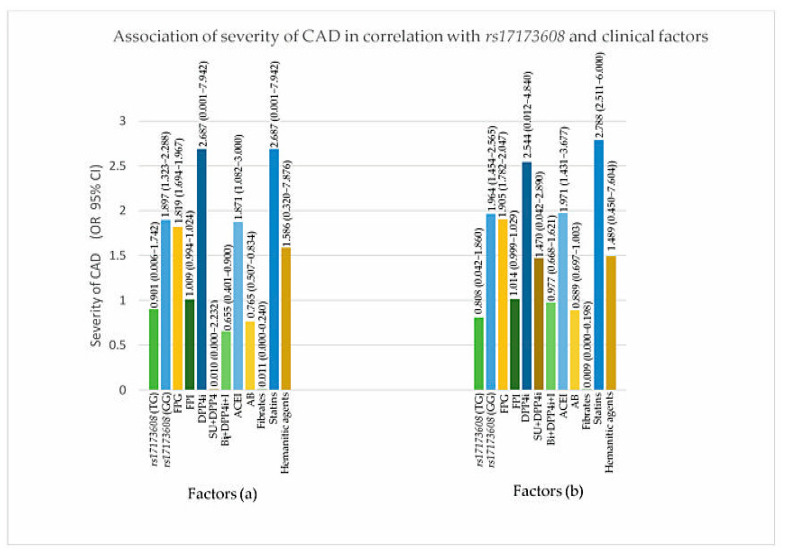
Association of severity of CAD in correlation with rs17173608 and clinical factors. (**a**) SVD vs. DVD, (**b**) SVD vs. TVD. FPG: fasting plasma glucose; FPI: fasting plasma insulin; AB: alpha blockers; ACEI: angiotensin-converting-enzyme inhibitor; DPP4i: dipeptidyl peptidase-4 inhibitor; I: insulin; SU: sulphonylureas.

From the secondary analysis, FPG (*p* value for IR = 0.047, DVD = 0.017, TVD = 0.032), ACEI (*p* value for IR = 0.024, DVD = 0.017, TVD = 0.025), *rs17173608* TG polymorphism (*p* value for IR = 0.015, DVD = 0.028, TVD = 0.036), and *rs17173608* GG polymorphism (*p* value for IR = 0.012, DVD = 0.042, TVD = 0.048) were associated with insulin resistance and the severity of CAD. Table 5 and Table 6, Figure 3 and Figure 4 show the secondary analysis of insulin resistance and the severity of CAD in correlation with *rs17173608* and clinical factors. Appendix A contains a summary of the findings of this study.

## 4. Discussion

Insulin resistance and the severity of CAD were postulated to come independently from “common soil”. The demographical and clinical factors associated with insulin resistance may be associated with CAD patients. The study by Arambewela et al. found that the prevalence of T2D with CAD was higher in males than females [29], and the study by Krishnan et al. found that the prevalence of CAD in non-diabetic CAD patients was higher in males than females [30]. Gender differences exist because, regardless of T2D, males develop CAD 7 to 10 years earlier than females [31]. Females are more likely to develop CAD as a result of massive hormonal changes following menopause [32].

Similarly, more males than females were found in T2D patients with CAD and CAD-only patients in our study. Nonetheless, females were diagnosed with T2D at a higher rate than males in T2D-only patients. The National Health and Morbidity Survey (NHMS) 2019 found that the prevalence of T2D was higher in females than in males [33]. Several studies have found that the prevalence of T2D and CAD varies across ethnic groups living in the same country. It has been postulated to be associated with environmental and genetic factors. Indian patients had the highest prevalence in our study, followed by Malay and Chinese patients in the T2D with CAD and T2D-only groups. Tee and Yap [34] discovered similar results in the Malaysian population [34]. This could be because Indians have a higher inherent risk of T2D and CAD in Malaysia [34].

FPG and A1C levels were higher in T2D patients with CAD than in T2D patients without CAD in a previous study [35]. As a result, the findings of the studies contradicted our findings. Meanwhile, LDL-c levels in T2D patients without CAD were higher than in T2D patients with CAD and CAD patients without T2D [35]. The results for FPI and HOMA-IR in the same study contradicted our findings, as the values were higher in T2D patients without CAD [35]. BMI is said to have an effect on glycaemic control in people with diabetes and other metabolic disorders. The BMIs of T2D-only patients were higher than the other two groups in this study, which may have resulted in an increase in FPG, A1C, LDL-c, FPI, and HOMA-IR levels in T2D patients only when compared to the other groups of patients.

Furthermore, previous studies were conducted on obese individuals, yielding contradictory results [35]. In a previous study, CAD patients had higher hs–CRP, HDL-c, and total cholesterol levels, which were comparable to the findings of our study. [36]. *rs17173608* is a *RARRES2* gene variant, and to the best of our knowledge, this is the first study to associate *rs17173608* with demographic and clinical factors of insulin resistance and severity of CAD. In the preliminary associations, some elements were discovered to be associated with *rs17173608*. Gender was associated with *rs17173608*. *rs17173608* was associated with increased visceral fat mass in non-obese subjects in one study [37]. Men tend to store more fat in their visceral depots. As a result, the association of *rs17173608* with the male gender is most likely due to gender-related body fat distribution [38]. Furthermore, according to Table 2, the majority of T2D patients with CAD were not receiving biguanide treatment (Yes = 47, No = 103).

As a result of the significant association of *rs17173608* with biguanides in preliminary analysis, it was hypothesised that the administration of biguanides to these patients would reduce the expression of *RARRES2* and thus chemerin. In the T2D-only group, however, many patients were on sulphonylureas. Sulphonylureas are the second-line drugs in T2D management, and the use is associated with an adverse vascular function, which results in endothelial dysfunction and cardiovascular events [39]. SNP *rs17173608* was associated with insulin resistance in our study and previous studies [36]. Besides this, *RARRES2* SNP *rs17173608* was associated with the severity of CAD in the study by Lachine et al. [40]. Our findings were consistent with previous studies, despite differences in the study population and pharmacological treatments. Secondary analyses were performed to determine the additional association of *rs17173608* polymorphisms (TT, TG, and GG) with insulin resistance and the severity of CAD in T2D patients with CAD. The secondary analysis revealed a significant association of *rs17173608* polymorphisms (TG and GG) with insulin resistance and the severity of CAD.

### Strength and Limitations

An essential strength of this study was the homogenous sample of individuals. All the samples were non-obese individuals. This enabled researchers to draw conclusions about the impact of *rs1717308* in a non-obese T2D population with and without CAD. Furthermore, this was the first study to associate *rs17173608* with both insulin resistance and the severity of CAD. The main constraint was the small sample size. Nonetheless, the samples were chosen using very strict inclusion and exclusion criteria in order to reduce the impact of confounding variables that could interfere with the outcome of this association study.

## 5. Conclusions

Among Malaysia’s three major ethnic groups, Indians had the highest prevalence of insulin resistance and the severity of CAD in the study population. The preliminary and secondary analyses revealed that the TG and GG polymorphisms of *rs17173608* were significantly associated with insulin resistance and the severity of CAD. Thus, the findings indicate that *rs17173608* is a significant predictor of insulin resistance and the severity of CAD.

## Figures and Tables

**Table 1:** Sample size calculation.

Outcome	:	Disease
Design	:	Case-control
Hypothesis	:	Gene only
Power	:	0.800
Significance	:	0.050000, two-sided
Gene	:	Mode of inheritance—log-additive
Pathogenic allele frequency	:	0.60

**Table 2:** Demographic and clinical factors of the study population (*n* = 300).

Factors	T2D + CAD *n* = 150	T2D *n* = 90	CAD *n* = 60	*p*-Value
Gender				
Male	^a^ 110 (73.3%)	^a^ 40 (44.4%)	^a^ 44 (73.3%)	<0.001 *
Female	^a^ 40 (26.7%)	^a^ 50 (55.6%)	^a^ 16 (26.7%)	
Age (years old)	^b^ 62.90 ± 8.63	^b^ 58.12 ± 8.48	^b^ 66.27 ± 10.28	**<0.001 ^¥^**
Ethnicity				
Malay	^a^ 57 (38.0%)	^a^ 35 (38.9%)	^a^ 17 (28.3%)	0.001 *
Chinese	^a^ 25 (16.7%)	^a^ 15 (16.7%)	^a^ 25 (41.7%)	
Indian	^a^ 68 (45.3%)	^a^ 40 (44.4%)	^a^ 18 (30.0%)	
BMI (kg/m^2^)	^b^ 25.23 ± 4.19	^b^ 25.38 ± 4.12	^b^ 24.95 ± 3.78	0.342 ^¥^
Family history of T2D	^a^ 99 (66.0%)	^a^ 73 (81.1%)	^a^ 40 (66.7%)	0.049 *
Family history of CAD	^a^ 85 (56.7%)	^a^ 44 (48.9%)	^a^ 39 (65.0%)	0.197 *
FPG (mmol/L)	^c^ 7.65 ± (6.00–9.93)	^c^ 8.15 ± (6.00–10.13)	^c^ 4.80 ± (4.33–5.38)	**<0.001 ^₺^**
FPI (pmol/L)	^c^ 27.40 ± (12.60–51.13)	^c^ 19.15 ± (10.58–33.33)	^c^ 30.90 ± (25.70–37.40)	**<0.001 ^₺^**
HOMA-IR	^c^ 9.80 ± (4.87–19.35)	^c^ 6.92 ± (4.35–11.56)	^c^ 6.73 ± (5.41–7.48)	**0.002 ^₺^**
A1C (%)	^c^ 7.50 ± (6.68–8.63)	^c^ 7.70 ± (6.40–9.50)	^c^ 4.70 ± (4.23–5.20)	**<0.001 ^₺^**
HsCRP (mg/L)	^c^ 0.50 ± (0.15–1.01)	^c^ 0.68 ± (0.25–1.07)	^c^ 0.93 ± (0.70–1.06)	**<0.001 ^₺^**
Lipid profile				
Total cholesterol (mmol/L)	^c^ 4.10 ± (3.40–5.00)	^c^ 4.31 ± (3.48–5.00)	^c^ 4.35 ± (3.73–4.88)	0.837 ^₺^
LDL-c (mmol/L)	^c^ 1.80 ± (1.40–2.51)	^c^ 2.52 ± (1.81–2.84)	^c^ 2.45 ± (1.77–2.92)	**<0.001 ^₺^**
HDL-c (mmol/L)	^c^ 1.30 ± (1.02–1.76)	^c^ 1.04 ± (0.88–1.36)	^c^ 1.08 ± (0.88–1.26)	**<0.001 ^₺^**
Triglycerides (mmol/L)	^c^ 1.50 ± (1.09–2.17)	^c^ 1.65 ± (1.30–2.30)	^c^ 1.75 ± (1.30–2.40)	**0.047 ^₺^**
Hypertension	^a^ 142 (94.7%)	^a^ 85 (94.4%)	^a^ 57 (95.0%)	1.000 ^Ᵹ^
Dyslipidaemia	^a^ 135 (90.0%)	^a^ 81 (90.0%)	^a^ 51 (85.0%)	0.572 ^Ᵹ^
Peripheral neuropathy	^a^ 59 (39.3%)	^a^ 40 (44.4%)	^a^ 0 (0.0%)	**<0.001 ^Ᵹ^**
Chronic kidney disease (CKD)	^a^ 26 (17.3%)	^a^ 18 (20.0%)	^a^ 2 (3.3%)	**0.012 ^Ᵹ^**
Retinopathy	^a^ 39 (26.0%)	^a^ 28 (31.1%)	^a^ 0 (0%)	**<0.001 ^Ᵹ^**
Anaemia	^a^ 2 (1.3%)	^a^ 0 (0.0%)	^a^ 2 (3.3%)	0.190 ^Ᵹ^
Gastritis	^a^ 0 (0.0%)	^a^ 4 (4.4%)	3 (5.0%)	**0.011 ^Ᵹ^**
Biguanides	^a^ 47 (31.3%)	^a^ 70 (77.8%)	-	<0.001 *
Sulphonylureas	^a^ 35 (23.3%)	^a^ 62 (68.9%)	-	<0.001 *
DPP4i	^a^ 1 (0.7%)	^a^ 26 (28.9%)	-	**<0.001 ^Ᵹ^**
AGI	^a^ 0 (0.0%)	^a^ 14 (15.6)	-	**<0.001 ^Ᵹ^**
Meglitinides	^a^ 0 (0.0%)	^a^ 14 (15.6%)	-	**<0.001 ^Ᵹ^**
Biguanide + SU	^a^ 26 (17.3%)	^a^ 63 (70.0%)	-	<0.001 *
SU + DPP4i	^a^ 1 (0.7%)	^a^ 5 (5.6%)	-	**0.035 ^Ᵹ^**
Biguanide + Insulin	^a^ 49 (32.7%)	-	-	**<0.001 ꟹ**
Biguanide + SU + Insulin	^a^ 8 (5.3%)	-	-	**<0.001 ꟹ**
Biguanide + DPP4i + Insulin	^a^ 5 (3.3%)	-	-	**<0.001 ꟹ**
Biguanide + SGLT2 + Insulin	^a^ 28 (18.7%)	-	-	**<0.001 ꟹ**
SGLT2 + Insulin	^a^ 5 (3.3%)	-	-	**<0.001 ꟹ**
Antiplatelet Agents	^a^ 144 (96.0%)	^a^ 0 (0.0%)	^a^ 59 (98.3%)	**<0.001 ^Ᵹ^**
ACEI	^a^ 83 (55.3%)	^a^ 22 (24.4%)	^a^ 12 (20.0%)	0.004 *
ARB II	^a^ 34 (22.7%)	^a^ 61 (67.8%)	^a^ 45 (75.0%)	0.087 *
Calcium channel blockers	^a^ 38 (25.3%)	^a^ 47 (52.2%)	^a^ 46 (76.7%)	0.220 *
Beta blockers	^a^ 82 (54.7%)	^a^ 47 (52.2%)	^a^ 10 (16.7%)	0.355 *
Alpha blockers	^a^ 3 (2.0%)	^a^ 0 (0.0%)	^a^ 0 (0.0%)	**0.001 ^Ᵹ^**
Nitrates	^a^ 52 (34.7%)	^a^ 0 (0.0%)	^a^ 53 (88.3%)	0.086 ^Ᵹ^
Statins	^a^ 149 (99.3%)	^a^ 88 (97.8%)	^a^ 60 (100.0%)	0.649 ^Ᵹ^
Diuretics	^a^ 51 (34.0%)	^a^ 28 (31.1%)	^a^ 53 (88.3%)	0.158 *
Haematinic agents	^a^ 4 (2.7%)	^a^ 3 (3.3%)	^a^ 7 (11.7%)	**0.028 ^Ᵹ^**
Cardiac glycosides	^a^ 13 (8.7%)	^a^ 0 (0.0%)	^a^ 0 (0.0%)	**0.001 ^Ᵹ^**

^a^ Nominal data are reported as numbers (percentages), whereas interval data are reported as ^b^ Mean ± SD (demographic data) or ^c^ Median ± IQR (in range 25th percentile-75th percentile) (clinical data); normally distributed: Mean ± SD, not normally distributed: Median ± IQR. * Calculated by using Chi-square; ^¥^ calculated by using one-way ANOVA; ^₺^ computed by using the Kruskal–Wallis test; ^Ᵹ^ computed using the Fisher exact test; ꟹ computed using one sample *T*-test. Bold font indicates significance at *p* < 0.05. ‘-’ indicates not relevant. ACEI: angiotensin-converting-enzyme inhibitor; AGI: alpha-glucosidase inhibitors; ARB II: angiotensin II receptor blockers; DPP4i: dipeptidyl peptidase-4 inhibitor; SGLT2: sodium-glucose co-transporter-2; SU: sulphonylureas.

**Table 3:** Associations of clinical factors with *rs17173608* in subjects (*n* = 300).

Clinical Factors	OR (95% CI)		
	T2D + CAD	T2D	CAD
FPG (mmol/L)	^a^ 0.921 (0.783–1.085) ^b^ 1.010 (0.906–1.127)	^a^ 0.923 (0.775–1.099) ^b^ 0.930 (0.790–1.095)	^a^ **3.646 (1.000–13.304)** ^b^ **6.053 (1.601–22.880)**
FPI (pmol/L)	^a^ 0.996 (0.986–1.007) ^b^ 0.993 (0.984–1.002)	^a^ 1.012 (0.980–1.046) ^b^ 1.014 (0.983–1.046)	^a^ **1.955 (1.854–2.068)** ^b^ **1.882 (1.787–1.990)**
A1C (%)	^a^ 0.984 (0.763–1.269) ^b^ 0.877 (0.708–1.086)	^a^ 0.847 (0.635–1.130) ^b^ 0.883 (0.677–1.150)	^a^ **3.822 (0.976–14.966)** ^b^ **5.360 (1.352–21.255)**
hs–CRP (mg/L)	^a^ 0.404 (0.136–1.200) ^b^ 1.034 (0.703–1.521)	^a^ 2.005 (0.558–7.210) ^b^ 2.708 (0.799–9.181)	^a^ 1.336 (0.203–8.792) ^b^ 5.517 (0.799–38.077)
Lipid profile TC (mmol/L)	^a^ 1.074 (0.746–1.545) ^b^ 0.799 (0.582–1.098)	^a^ 1.091 (0.650–1.829) ^b^ 0.893 (0.544–1.467)	^a^ 0.625 (0.304–1.284) ^b^ 0.823 (0.424–1.597)
LDL-c (mmol/L)	^a^ 1.576 (0.954–2.602) ^b^ 1.189 (0.778–1.818)	^a^ 1.291 (0.606–2.752) ^b^ 1.045 (0.503–2.170)	^a^ 0.619 (0.245–1.565) ^b^ 0.995 (0.419–2.363)
HDL-c (mmol/L)	^a^ 1.209 (0.720–2.029) ^b^ 0.993 (0.638–1.546)	^a^ 1.591 (0.188–13.434) ^b^ 0.400 (0.050–3.192)	^a^ 0.311 (0.017–5.663) ^b^ 0.276 (0.016–4.696)
Triglycerides (mmol/L)	^a^ 1.293 (0.802–2.085) ^b^ 0.798 (0.509–1.251)	^a^ 1.842 (0.795–4.268) ^b^ 1.390 (0.612–3.161)	^a^ 0.656 (0.263–1.635) ^b^ 1.029 (0.449–2.359)
Hypertension	^a^ 1.975 (0.337–11.559) ^b^ 0.919 (0.162–5.221)	^a^ 4.735 (0.742–10.343) ^b^ 4.850 (0.960–8.066)	^a^ 1.307 (0.700–14.434) ^b^ 1.403 (0.817–12.235)
Dyslipidaemia	^a^ 0.882 (0.164–4.113) ^b^ 0.802 (0.223–2.767)	^a^ 1.071 (0.090–12.807) ^b^ 2.368 (0.262–21.370)	^a^ 0.333 (0.039–2.829) ^b^ 0.761 (0.120–4.816)
Peripheral neuropathy	^a^ 2.871 (0.969–8.511) ^b^ 1.531 (0.725–3.233)	^a^ 1.143 (0.339–3.850) ^b^ 1.444 (0.458–4.560)	^a^ 0.100 (0.076–0.323) ^b^ 0.098 (0.082–0.285)
Chronic kidney disease (CKD)	^a^ 0.690 (0.218–2.180) ^b^ 1.101 (0.409–2.964)	^a^ 1.154 (0.238–5.605) ^b^ 0.785 (0.186–3.311)	^a^ 1.317 (0.412–43.004) ^b^ 3.375 (0.189–60.238)
Retinopathy	^a^ 0.872 (0.316–2.405) ^b^ 1.881 (0.765–4.625)	^a^ 0.399 (0.093–1.714) ^b^ 0.495 (0.121–2.019)	^a^ 0.204 (0.098–1.063) ^b^ 0.188 (0.094–1.007)
Anaemia	^a^ 0.476 (0.046–6.406) ^b^ 0.537 (0.033–8.788)	-	^a^ 2.750 (0.153–49.359) ^b^ 1.337 (0.877–34.435)
Gastritis	-	^a^ 4.472 (0.855–9.031) ^b^ 6.708 (0.652–14.454)	-
Single-OHA			
Biguanides	^a^ 0.552 (0.194–1.567) ^b^ **1.246 (1.112–1.543)**	^a^ 0.185 (0.039–0.885) ^b^ 0.556 (0.164–1.883)	-
Sulphonylureas	^a^ 0.541 (0.180–1.626) ^b^ 0.406 (0.174–0.945)	^a^**8.000 (1.542–41.413)** ^b^ 2.059 (0.399–10.622)	-
DPP4i	^a^ 0.986 (0.730–1.269) ^b^ 0.014 (0.008–0.040)	^a^ 2.556 (0.696–9.382) ^b^ 2.074 (0.631–6.817)	-
OHA-Combination			
Biguanide + SU	^a^ 0.466 (0.141–1.541) ^b^ 0.423 (0.164–1.093)	^a^ 2.889 (0.676–12.345) ^b^ 1.625 (0.393–6.722)	-
SU + DPP4i	^a^ 0.955 (0.890–1.112) ^b^ 0.020 (0.008–0.057)	^a^ 1.236 (0.102–3.805) ^b^ 1.952 (0.295–12.914)	-
OHA- Insulin Combination			
Biguanide + Insulin	^a^ 1.487 (0.548–4.034) ^b^ **3.209 (1.330–7.741)**	-	-
Biguanide + SU + Insulin	^a^ 1.636 (0.187–14.350) ^b^ 3.429 (0.400–29.409)	-	-
Biguanide + DPP4i + Insulin	^a^ 0.122 (0.011–1.413) ^b^ 0.262 (0.023–2.974)	-	-
Biguanide + SGLT2 + Insulin	^a^ 1.754 (0.466–6.597) ^b^ 1.503 (0.575–3.928)	-	-
SGLT2 + Insulin	^a^ 0.787 (0.078–7.962) ^b^ 1.650 (0.167–16.341)	-	-
Concomitant drugs			
Antiplatelet Agents	^a^ 8.200 (0.708–94.997) ^b^ 5.857 (0.591–58.043)	-	^a^ 3.060 (2.063–11.533) ^b^ 1.020 (0.845–1.480)
ACEI	^a^ 1.492 (0.580–3.835) ^b^ 0.829 (0.397–1.732)	^a^ 0.778 (0.197–3.076) ^b^ 1.296 (0.337–4.990)	^a^ **2.375 (1.410–13.748)** ^b^ **2.300 (1.424–12.465)**
ARB II	^a^ 0.636 (0.226–1.787) ^b^ 1.373 (0.547–3.445)	^a^ 2.000 (0.520–7.591) ^b^ 1.258 (0.342–4.634)	^a^ 2.375 (0.410–13.748) ^b^ 1.250 (0.250–6.255)
Calcium channel blockers	^a^ 0.520 (0.190–1.424) ^b^ 0.918 (0.392–2.149)	^a^**7.485 (1.741–32.183)** ^b^ 3.957 (0.988–15.850)	^a^ 0.421 (0.073–2.437) ^b^ 0.667 (0.131–3.398)
Beta blockers	^a^ 0.746 (0.283–1.970) ^b^ 1.492 (0.720–3.093)	^a^ 0.400 (0.115–1.394) ^b^ 0.548 (0.170–1.769)	^a^ **3.000 (1.353–25.460)** ^b^ **1.848 (1.171–6.416)**
Alpha blockers	^a^ 1.635 (0.268–4.231) ^b^ 1.086 (0.096–12.320)	-	-
Nitrates	^a^ 0.735 (0.280–1.928) ^b^ 1.018 (0.472–2.197)	-	^a^ 0.333 (0.039–2.829) ^b^ 0.420 (0.058–3.029)
Fibrates	^a^ 4.505 (0.650–15.369) ^b^ 4.508 (0.684–15.026)	-	-
Statins	^a^ 0.030 (0.009–0.453) ^b^ 0.032 (0.012–0.086)	^a^ 0.058 (0.035–0.097) ^b^ 0.039 (0.013–0.831)	-
Diuretics	^a^ 0.611 (0.235–1.587) ^b^ 1.253 (0.569–2.759)	^a^ 1.971 (0.527–7.374) ^b^ 1.160 (0.353–3.808)	^a^ 0.159 (0.012–2.031) ^b^ 0.583 (0.088–3.880)
Haematinic agents	^a^ 13.109 (0.995–20.246) ^b^ 0.531 (0.072–3.901)	^a^ 0.032 (0.018–0.153) ^b^ 1.400 (0.118–16.581)	^a^ 3.000 (0.353–25.460) ^b^ 2.381 (0.330–17.172)
Cardiac glycosides	^a^ 1.636 (0.187–14.350) ^b^ 0.506 (0.153–1.674)	-	-

Computed using multinomial logistic regression analysis. Bold font indicates significance at *p* < 0.05. OR: odds ratio; CI: confidence interval. *rs17173608*: ^a^ TT vs. GG, ^b^ TG vs. GG. Adjusted for covariates age, race, gender, and BMI. ‘-’ indicates not relevant. FPG: fasting plasma glucose; FPI: fasting plasma insulin; A1C: glycated-haemoglobin; hs–CRP: high-sensitive C-reactive protein; LDL-c: low-density lipoprotein cholesterol; HDL-c: high-density lipoprotein cholesterol; TC: total cholesterol; ACEI: angiotensin-converting-enzyme inhibitor; AGI: alpha-glucosidase inhibitors; ARB II: angiotensin II receptor blockers; DPP4i: dipeptidyl peptidase-4 inhibitor; SGLT2: sodium-glucose co-transporter-2; SU: sulphonylureas.

**Table 4:** Associations of *rs17173608* with insulin resistance and severity of CAD.

	OR (95%CI)		
	T2D + CAD	T2D	CAD
Insulin resistance	1.861 (1.330–2.247) *	1.458 (0.422–5.042) *	1.435 (1.080–3.356) *
Severity of CAD	**^a^ 1.783 (1.335–2.409) ^¥^** **^b^ 1.822 (1.359–2.870) ^¥^**	-	**^a^ 1.905 (1.045–2.866) ^¥^** **^b^ 1.634 (1.322–1.989) ^¥^**

* Computed using binary logistic regression analysis. **^¥^** Computed using multinomial logistic regression analysis. HOMA-IR cut-off point: 7.17. Bold font indicates significance at *p* < 0.05. Insulin-sensitive (IS) used as the reference group (IS vs. IR) for insulin resistance. SVD was used as the reference group (^a^ SVD vs. DVD, ^b^ SVD vs. TVD) for severity of CAD. Adjusted for the covariates age, race, gender, and BMI. OR: odds ratio; CI: confidence interval. ‘-’ indicates not relevant.

**Table 5:** Associations of insulin resistance in correlation with *rs17173608* and clinical factors.

Parameters	OR (95% Cl)	*p*-Value
*rs17173608*		
TT		0.157
TG	0.802 (0.055–11.592)	**0.015**
GG	1.680 (1.130–2.772)	**0.012**
FPG (mmol/L)	1.151 (1.013–1.309)	**0.047**
FPI (pmol/L)	1.004 (0.949–1.063)	0.676
hs–CRP	2.687 (0.001–7.942)	0.808
Biguanide + DPP4i + Insulin	1.480 (0.054–4.032)	0.607
Antiplatelet Agents	1.064 (0.087–2.543)	0.213
ACEI	2.050 (0.063–6.633)	**0.024**

A binary logistics regression test was done to obtain the OR and *p* values. Insulin sensitive (IS) was used as the reference group (IS vs. IR). Adjusted for covariates age, race, gender, and BMI. OR: odds ratio; CI: confidence interval. Bold font indicates significance at *p* < 0.05. FPG: fasting plasma glucose; FPI: fasting plasma insulin; hs–CRP: high-sensitive C-reactive protein; ACEI: angiotensin-converting-enzyme inhibitor; DPP4i: dipeptidyl peptidase-4 inhibitor.

**Table 6:** Associations of severity of CAD in correlation with *rs17173608* and clinical factors.

Parameters	DVD		TVD	
	OR (95% CI)	*p*-Value	OR (95% CI)	*p*-Value
*rs17173608*				
TT		0.052		0.122
TG	0.901 (0.006–1.742)	**0.028**	0.808 (0.042–1.860)	**0.036**
GG	1.897 (1.323–2.288)	**0.042**	1.964 (1.454–2.565)	**0.048**
FPG (mmol/L)	1.819 (1.694–1.967)	**0.017**	1.905 (1.782–2.047)	**0.032**
FPI (pmol/L)	1.009 (0.994–1.024)	0.255	1.014 (0.999–1.029)	0.074
DPP4i	2.687 (0.001–7.942)	0.808	2.544 (0.012–4.840)	0.656
SU + DPP4i	0.010 (0.000–2.232)	0.096	1.470 (0.042–2.890)	0.996
Biguanide + DPP4i + Insulin	0.655 (0.401–0.900)	0.995	0.977 (0.668–1.621)	0.080
ACEI	1.871 (1.082–3.000)	**0.017**	1.971 (1.431–3.677)	**0.025**
Alpha-blockers	0.765 (0.507–0.834)	0.990	0.889 (0.697–1.003)	0.989
Fibrates	0.011 (0.000–0.240)	0.996	0.009 (0.000–0.198)	0.992
Statins	2.687 (0.001–7.942)	0.808	2.788 (2.511–6.000)	0.988
Hematinic agents	1.586 (0.320–7.876)	0.090	1.489 (0.450–7.604)	0.105

Multinomial logistics regression test was done to obtain the OR and *p* value. SVD was used as the reference group (DVD: SVD vs. DVD, TVD: SVD vs. TVD). Adjusted for covariates age, race, gender, and BMI. Bold font indicates significance at *p* < 0.05. OR: odds ratio; CI: confidence interval. Bold font indicates significance at *p* < 0.05. FPG: Fasting plasma glucose; FPI: fasting plasma insulin; ACEI: angiotensin-converting-enzyme inhibitor; DPP4i: dipeptidyl peptidase-4 inhibitor; SU: sulphonylureas.

## Data Availability

The data presented in this study are available on request from the corresponding author. The data are not publicly available as they contain the information that could compromise the privacy of research participants.

## Appendix A

**Table A1:** Summary points.

**Chemerin and *RARRES2*** Chemerin and its coding gene *RARRES2*, involved in the pathogenesis of metabolic syndrome, induce angiogenesis and activate an inflammatory response. The increased level of chemerin and expression of *RARRES2* results in insulin resistance, leading to T2D and atherosclerotic CAD. Several studies have been conducted on the association of SNP *rs17173608* with insulin resistance or atherosclerotic CAD.
**Insulin resistance and severity of CAD** Endothelial dysfunction is the main culprit that results in insulin resistance and progression of atherosclerosis. The CAD is severe when the atherosclerotic plaques narrow down the vessels with more than 50% of stenosis. In our study, we included the patients with more than 50% of atherosclerotic stenosis, and we classified the patients as having SVD, DVD, and TVD (the most severe type of CAD)
**Demographic and clinical factors** Age, gender, ethnicity, BMI, and family history of T2DM and CAD were the demographic factors assessed in our study. Indians had the highest prevalence of insulin resistance and severity of CAD than Malays and Chinese. Laboratory investigations, comorbidities, and pharmacological treatments were the clinical factors we studied. We excluded some of the comorbidities such as obesity and asthma, as these diseases may influence the outcome of the study.
***rs17173608*** *rs17173608* is a SNP of *RARRES2* gene. Some studies showed a significant correlation between *rs17173608* and T2D whilst a significant proportion of previous evidence exhibiting a correlation between *rs17173608* and CAD. Our study is the first study explicitly associating *rs17173608* with both insulin resistance and severity of CAD in non-obese T2D patients.
**Conclusion** TG and GG polymorphisms of *rs17173608* were significantly associated with insulin resistance and the severity of CAD. *rs17173608* is a significant predictor of insulin resistance and the severity of CAD in non-obese T2D patients.
